# Critical points in the experience of spouse caregivers of patients who have suffered a stroke. A phenomenological interpretive study

**DOI:** 10.1371/journal.pone.0195190

**Published:** 2018-04-04

**Authors:** Fidel López-Espuela, Teresa González-Gil, Javier Amarilla-Donoso, Sergio Cordovilla-Guardia, Juan Carlos Portilla-Cuenca, Ignacio Casado-Naranjo

**Affiliations:** 1 Department of Nursing, University of Extremadura, Facultad de Enfermería y Terapia Ocupacional, Cáceres, Spain; 2 Nursing Section Department, Faculty of Medicine, Universidad Autónoma de Madrid, Madrid, Spain; 3 Department of Nursing, Hospital Campo Arañuelo, Navalmoral de la Mata, Cáceres, Spain; 4 Stroke Unit, Hospital San Pedro de Alcántara, Cáceres, Spain; DEFACTUM, DENMARK

## Abstract

**Aims:**

To explore and document the experiences and values of spouse caregivers of stroke survivors. To gain more in-depth knowledge of how the act of caring and the adaption process affects caregiving spouses.

**Materials and methods:**

Phenomenological, qualitative study. This study included spouses of stroke survivors who also served as primary caregivers. Individual, semi-structured, in-depth interviews were conducted, transcribed, and analysed using a thematic content analysis as proposed by Giorgi. Data was analysed and managed using Atlas-ti (version 7.0). This study was approved by our institution´s Complejo Hospitalario de Caceres Ethics and Research Committee.

**Results:**

Spouse caregivers of stroke survivors undergo a process of disruption in their private lives and relationships, marked by their caregiving duties. The experiences of spouses caring for stroke survivors is condensed into topics and subtopics: “*Caregiving and that´s all”* summarizes the sense of having no life horizons and also caregiver impossibility of moving away from caring role. Otherwise, “*Breaking the couple´s life together”* enlightens the further consequences of stroke in couples shared live biography, which needs to be understood and rebuilt. Finally, “*Going through the (non) loss alone”* alludes to how spouses reinterpret meaning of loss, which is not appreciated by others and that refers to the balance between stroke survival and any acquired global disability.

**Conclusions:**

A comprehensive approach to the couple (family), from a psycho-socio-emotional and relational perspective, is essential for ensuring adequate quality of life for people who suffered a stroke as well as their caregivers. Spouses-caregivers must be considered as individuals living a transition process due to their unexpected disrupting change, which nurses should address through a comprehensive and integrated approach focused on transition care. Care resources must be adapted to the interpretation that the spouses have of their caregiving role and their relationship with the different components of the caregiving process.

## Introduction

Stroke is a first-order health problem; it is the second leading cause of death in the world and the primary cause of disability in adults. Stroke significantly impacts longevity [1,2] and is associated with a number of health problems [3].

In the last few years, the role of the informal caregiver has become a key political, social and economic issue in terms of cost-effectiveness [4–6]. The National Alliance for Caregiving has drawn special attention to caregivers as a vulnerable group, identifying caregiving overload and caring complexity as causes of emotional stress, financial strain, and health decline [7]. Caregiving knowledge overload and caring complexity, encourages a commitment to research as well as to develop evidence based interventions [4,8].

The subjective experience of surviving a stroke is characterised by a radical change in the lives of patients and their families [9], impacting physical, psychological, social, relational, emotional and spiritual experiences [1,10,11]. Everyday situations that were carried out with normalcy before stroke become now a real challenge for family members [1]. Furthermore, this dependence occurs abruptly and suddenly, and from one day to the next, close relatives become responsible of taking care of the dependent. In most cases, the spouse becomes the caregiver [9,12,13].

Previous research suggests that care-givers can, frequently, develop some problems such as pain, fatigue, loneliness, depression and poor health outcomes [14], which may affect well-being, life satisfaction, quality of life and mood [13,15]. Simultaneously, this responsibility can also bring positive experiences, such a more purposeful life, greater inner strength, learning new skills (medical/nursing tasks) or a greater closeness with the patient [16–18].

The caregiver role and its impact on health has been studied from different viewpoints and methodological strategies [1,4,5,9,19,20]. Although some studies focus on the perspective of the caregiver of a stroke survivor [15], only a few have specifically focused on spousal experiences and marital relationships [9, 21] being no evidence analyzed in Spain. Among them, it is interesting to highlight the meta-synthesis by Quinn et al. [13], that illustrates how spouses cope and adapt to caregiving when their partner has a stroke, identifying barriers and unmet needs and describing some coping strategies such as: “seeking information”, “searching for own space and well-being”, “suffering in silence”, “putting one’s own needs aside”, “adapting to a changed role”, looking for “social support” or adopting “hope and optimism”. Some derived clinical practice recommendations suggest that: the appropriateness of educational individual and group programs, the provision of respite care and cognitive behavioural therapy. Nevertheless, these studies must be expanded with some experimental research.

Literature emphasises the importance of physical and cognitive consequences of stroke, which are deeper perceived by spouses and constitute a long-term challenge for the couple [22]. Both alterations contribute to stroke survivor loss of self [23] and biographical disruption [24], which directly affect those surrounding the chronically ill. Spouses are primarily affected, putting marital relations in a vulnerable situation [9, 13] and making caregiving a difficult task.

Meleis A.I. [25, 26] proposes, that the process of coping with a chronic issue may be researched under the Transitions Theory framework. Various authors support this scheme i.e Messias and his work [27] on the experiences of parents of children diagnosed with congenital heart disease and the doctoral research of Schumacher [28] that focused on the acquisition of the role of family caregiver of persons receiving chemotherapy. Furthermore, Levine [16], focusing on management and policy strategies to enhance family long-term caregiving, identified different intervention programmes on the role of transitional care nurse. Transitions Theory offers then, a suitable framework to deal with abrupt acquired chronic processes and their consequences. Consequently, caregivers must be considered as a vulnerable population facing a health/illness transition. At the same time, nurses are expected to be competent to support them along transition, promoting facilitators and reducing inhibitors to promote healthy transition.

The present research aims to explore the experiences of spouse caregivers of patients who suffered a stroke, and to gain more in-depth knowledge about the needs and demands of spouses to facilitate the transition to caregiving [26].

## Materials and methods

To achieve these research aims, a qualitative methodology was undertaken with an interpretive phenomenological approach [29]. Phenomenological research was used to answer questions of meaning trying to understand the experience as is interpreted by those living such experience. We pretend to study how spouses interpret their lives and make understanding of their experience of caring their love one after a stroke.

The fieldwork was developed with spouses of patients who were admitted to the Stroke Unit of a five hundred beds Secondary Hospital of the Spanish Public Health System Network from June 2012 to January 2014. The Stroke Unit has ten-year clinical trajectory of operating with an integral perspective and it is provided with resources to assure care continuity from the stroke event to patient comprehensive rehabilitation. During its operating period, more than three thousand patients were treated, and low long term physical damages were observed in 60% of them.

The following population inclusion criteria were considered: spouses of patients who suffered a stroke and survived, spouses who undertook the role of main caregiver for more than 6 months after discharge [5], and spouses who voluntarily agreed to take part in the study. It was considered that 6 months after discharge would be enough time for caregivers to reflect about their caring experience providing information about their transition along the caring process [30,31]. Purposive sampling considered the spouse age, length of caregiving time and stroke survivors’ acquired incapacity aiming to select a sample that comprised heterogeneous profiles [32].

Nineteen caregivers were recruited in the context of follow-up nursing consulting room where they received information related to the study. Furthermore, they were given a document with written information to calmly read at home in order to reinforce knowledge about the study. A week later, researchers phoned them to clarify information and solve doubts as well as confirm their acceptance to participate and arrange an interview. None of the caregivers declined participating. They all signed the written consent and agreed interview audio recording. One spouse was interviewed but her discourse was not included in the analysis because she was divorced and, as a consequence, was not developing the role of caregiver at the moment of the interview.

The final sample consisted of 18 spouses (5 male spouse and 13 female spouse) whose contributions resulted in a saturation of themes and subthemes. Right after, we show some characteristics of the participants, which were obtained before the in-depth interview, by a socio-demographic questionnaire and some other later mentioned tools were completed. Spouses included in the sample are at an average age of 55 years (SD: 10.63), and in some cases, they combined care with an active working life (33%). They have been developing a caregiver role during an estimated time of 28.72 months (SD: 25.98), only receiving socio-economic help in 30% of the cases. In general, the health of caregivers was subjectively considered as “good” (measured by a 5 items Likert Scale: very bad-bad, normal, good, very good), although they reported suffering from mood swings, with a tendency towards low mood in 61% of participants. In terms of caregiver overload, according to the Zarit Burden Interview [33], 5 of the 18 caregivers exhibited moderate or severe overload. Most stroke survivors were independent or mildly dependent on caregivers for basic daily activities and had higher levels of dependence in instrumental daily activities (taking Barthel Index and Lawton and Brody Scale as reference) [34, 35] (Table 1). There were some limitations in sampling (Table 1); i.e., it was difficult to access male caregivers, extreme or negative cases related to age (<40 years old), and cases with greater levels of dependency (total or severe).

**Table 1:** Sample characteristics.

Code	Spouse-caregiver characteristics											Stroke survivor characteristics		
	Caregiver age	Caregiver relationship with stroke survivor	EmploymentSituation	Income per Month	Hours of Care (per Day)	Time of Care (Months)	Family Relationship	Social Aid	Zarit Burden Interview	Depressed mood	Subjective Perception of Health	Age	Lawton & Brody Scale	BarthelIndex
1	67	Husband	Retired	1000–1500€	24	33	Excellent	Yes	Mild Burden	Yes	Good	65	1	50
2	53	Wife	Housewife	>1500€	24	60	Good	No	Mild Burden	Yes	Good	56	4	100
3	80	Husband	Retired	>1500€	24	102	Very Good	No	Mild Burden	Yes	Good	78	2	80
4	42	Wife	Employed	>1500€	16	15	Very Good	No	Mild Burden	Yes	Very Good	43	6	100
5	49	Husband	Employed	500–1000€	24	15	Very Good	No	Minimal Burden	Yes	Regular	50	2	80
6	58	Wife	Housewife	1000–1500€	24	25	Very Good	Yes	Minimal Burden	Yes	Good	60	4	60
7	56	Wife	Housewife	1000–1500€	24	13	Regular	Yes	Minimal Burden	Yes	Regular	60	3	15
8	56	Wife	SickLeave	>1500€	4	48	Good	No	Minimal Burden	Yes	Regular	59	1	70
9	54	Husband	Employed	>1500€	8	30	Good	No	Minimal Burden	No	Good	62	4	95
10	42	Wife	Housewife	1000–1500€	24	36	Very Good	Yes	Minimal Burden	No	Good	48	4	85
11	53	Wife	Housewife	1000–1500€	24	68	Regular	No	Minimal Burden	Yes	Poor	43	3	95
12	70	Husband	Retired	>1500€	24	14	Very Good	Yes	Severe Burden	No	Good	57	2	70
13	62	Wife	Retired	500 Y 1000€	24	10	Excellent	No	Mild Burden	No	Very Good	70	3	55
14	70	Wife	Retired	>1500€	24	15	Good	No	Moderate Burden	No	Regular	70	3	70
15	45	Wife	Housewife	1000–1500€	24	6	Regular	No	Moderate Burden	No	Excellent	45	6	100
16	50	Wife	Employed	>1500€	18	11	Good	No	Moderate Burden	No	Good	52	1	60
17	47	Wife	Unemployed	>1500€	24	5	Regular	No	Moderate Burden	No	Very Good	55	7	100
18	60	Wife	Employed	>1500€	24	6	Very Good	No	Mild Burden	Yes	Regular	66	4	75

Lawton & Brody Scale: 0- dependency; 8- independence.

Barthel Index: total dependency (<20 points), severe dependency (21–40), moderate dependency (41–60), mild dependency (61–90) and independence (91–100).

Information was collected via in-depth individual and semi structured interviews [36] using a dynamic interview script that kept evolving and focusing on emerging issues as researchers moved ahead in the data analysis phase (Table 2). Interviews were conducted in a neurology office by a single interviewer (a nurse who is a member of the research team and was previously trained in the technique), lasting from 80 to 110 minutes, which also included the time for completing the previously mentioned survey (socio-demographic one, Lawton & Brody Scale, Barthel Index and Zarit Burden Interview). In some interviews, it was difficult to go through an in-depth experience disclosure. Such limitation could be related to participant’s lack of introspection or interviewer´s difficulties to stablish a good rapport. Furthermore, conducting the interviews in the same clinical setting where families had gone through their acute post-stroke experience may have established a confusing relationship between researcher and participant (therapeutic against researcher role), by influencing participant´s subsequent comments. All interviews were recorded in audio format and transcribed for further analysis. During several interviews, participants emotionally collapsed, making it necessary to quit and redirect the interview.

**Table 2:** Interview guide.

INITIAL INTERVIEW GUIDE	Describe what happened the day before and after the stroke.
	How were the early days like: the moment of diagnosis and the first weeks at home?
	Being a caregiver may have involved doing activities which you were not use to do before, such as feeding or bathing your loved one. How did you experience these situations and how do you deal with them now? How about your loved one?
	How do you feel in this dependency situation? How did you adapt to this caregiving situation? How do you deal with your role as a caregiver? And how about your loved one?
	What aid resources have you had? Where you provided with any aid resources? Which one? What aid resources would you like to have?
	How has your life changed since you are the family caregiver? What do you expect from the future?
ADVANCED INTERVIEW GUIDE	What priority does care for your loved one have in your life?
	How do you see your life of caring for your loved one?
	How would you describe the life you used to have and the life you have now?
	How do you approach the future?
	What do you expect from life?
	What was your experience during the first months of caring your loved one?
	How did you adapt to caring your loved one? How did you adquired caring skills? How did you adjust to the environment and acquire caregiving resources?
	What type of assistance did you receive to be trained as a caregiver?
	As of today, to what degree do you see yourself capable of caring for your partner?
	How do you see delegating care to a professional caregiver?
	What type of aid do you believe might be appropriate for your care demand needs?
	What do you think of care assistance?
	What do you think of your partner as opposed to what he/she was before the stroke? What differences can you appreciate in your partner before and after the stroke?
	How do you think you have changed? Do you see yourself differently? How? Do you recognise yourself? What do you think of the process of reconnecting with whom you were before?
	How has surviving the stroke affected your life positively or negatively (for you and your partner)?
	What have you loose after the stroke?
	What positive things you want to point out after the stroke?

For data analysis, a thematic content analysis was carried out following the scheme proposed by Giorgi [37]. First, the interview transcription was read and re-read to identify significant information in response to the research questions and study objectives. Once data were highlighted, significant units were identified and coded according to the suggested meaning determined by the analyst researcher. Codes were all defined in a glossary, which allowed an initial interpretation. These codes were regrouped, generating themes and subthemes. Grouping was based on the shared meaning of codes and facilitated by comparing attitude and relationship linking (through networks diagramming). Theoretical reflective writing was a constant during this process [38]. Finally, in an interpretative exercise aiming to integrate all themes and subthemes, a central theme emerged, which encapsulates the essence of the experience. Attending to data saturation, we would like to add that the sense of *results showing an integral and assembled proposal*, and *researchers deep understanding of phenomena in coherence with research question* were key reference statements. The Atlas-ti version 7.0 programme was used to assist in data analysis.

The criteria of credibility, transferability and confirmability, as proposed by Guba and Lincoln [39], have been present throughout the study. Various strategies were developed to ensure credibility such as: reflexivity (with a constant reflective attitude attending methodological crossroads decision making and interpretative issues), research triangulation (comparison and discussion between researchers during analytical process) and return to interviewees (during the last interviews, as themes were becoming saturated, new questions were introduced in interviews to confirm the intersubjective experience interpretation) and clinical experts (some colleagues with wide clinical experience in the Stroke Unit critically read the results trying to provide commentaries and suggestions about no clear statements) [40]. In relation with confirmability, along this manuscript we tried to be transparent with our research process and other show researchers different stages and how we coped with different tasks and difficulties. Finally, in terms of helping audience to assess results transferability, we meticulously described the participants characteristics that conditions their experience as well as the environment were the research was developed.

This study was approved by the Ethics and Research Committee of Hospital and, during its implementation, the work team ensured the ethical principles of autonomy (informed consent), beneficence and non-maleficence (data confidentiality and care for emotional vulnerability) and equity (theoretical sampling in advanced stages of the analysis process).

## Results

In terms of needs and demands to facilitate the transition to caregiving, the experience of spouses is interpreted as: *Being aware and visualising critical points in caregiving*, which is condensed into three major themes that emerged from participant´s narratives (Table 3): (1) Intensity of caregiving, labelled "caregiving and that’s all", (2) Disruption in the couple, labelled "breaking the couple’s life" and (3) Loneliness of caregiving, labelled "going through the non-loss alone”. *Caregiving and that´s all* summarizes the sense of having no life horizons and the caregiver impossibility of moving away from the caring role. Otherwise, *Breaking the couple´s life together* enlighten the further consequences of stroke in couples shared live biography, so that the meaning of loss is reinterpreted as the other acquired disability not visible for others except the caregiver who suffers loneliness (*Going through the (non)loss alone*).

**Table 3:** Critical points identified by caregivers.

The experiences of spouse caregivers of patients who have suffered a stroke in terms of needs and demands to facilitate the transition to caregiving are condense in the idea of:		
BEING AWARE AND VISUALISING CRITICAL POINTS IN CAREGIVING		
Being Critical Points the following:		
“CAREGIVING AND THAT´S ALL”	“BREAKING THE COUPLE´S LIFE TOGETHER”	“GOING THROUGH THE (NON) LOSS ALONE”
Is living…	Is living…	Is living…
… A “***Life without horizons***”, a “***New non-life”***, with ***“Dark future”***.	*…Coping with my spouse as “****someone else****” (different to one he/she was before)*, *with “****me as caregiver****” (having lost my previous identity) and with a “****new relationship****” between us*.	… Facing ***“the caregiver´s acquired disabilit****y”*.
… Feeling “***Stuck to the role of caregiver***” with ***“Attachment and persistence in care”***, and ***“Short breaks needed”***.	***…*** Working hard for ***“Seeking for the essence of the other”*** and ***“Reconnecting”***.	… ***“Crying in loneliness***”.

### Caregiving and that’s all

With respect to *Caregiving and that’s all*, caregivers refer to the fact that their lives focus exclusively on the care of the dependent person. They feel forced to leave behind a life they considered *normal* to switch to a radically different life that participants define literally as *non-life* (life that may not be considered dignified). They describe caring for their partner as the centre around which everything moves; everything boils down to caring for the stroke survivor. *Caregiving and that’s all* has a negative meaning in the sense that it *ruins one’s life*. In this sense, some interviewees refer to their life as *the non-life I live* or the *life I’ve stopped living*, which suggest that, after the critical event of stroke and its consequences (dependence and caregiving), they are in a disconnected and unstable early transition phase. Dependence and caregiving may be interpreted, consequently, as disruptive events that come abruptly into one´s life destroying previous life and establishing a new life dynamic that does not suits with future expectations and life meaning.

***C6:*** “*So what is my life, then? Nothing. I now live to take care of my husband because it’s what I want, and that’s it*…”

***C8:*** “… *I feel young and I can do other things… but we are two prisoners at home. Our life is at home… I am taking care of him all day, and that is our life… I’d like to go out… and live another life*…”

In any case, this *New fatal life* or the *New non-life* (as most participants refer to) embodies a *Dark future* perspective that extends over the work, relational and personal development landscape and affects their life projection. Rupture with past life and future life expectations leads to a biographical disruption. Only those caregivers who were aware of the irreversible changes and who engaged with the transition process would have started to develop effective coping strategies.

***C1:*** “… *because I did many things. I have had to throw away my job, which is not very nice. So then, your horizon looks bleak. All you have left is death, so thoughts of death will no doubt emerge. It is best not to give it too much thought. Yes, life is not worth living like this. This life is no longer life. We’ll see what we can get out of it, but it’s not life… don’t be scared*”.

***C16:*** “*The stroke has turned my life on its head… How do I move forward with this now?… It’s hard, difficult, but it is something that I can handle*…”

Involved in this dynamic of *caregiving and that’s all*, an attitude of a*ttachment and persistence in care* is identified. This attachment is interpreted in two different ways:

First, it refers to the resilience in coping with that care. Although interviewees express different types of motivation to deal with care (love for the other, moral obligation, social pressure), there is a common tendency to recognise the other’s care as their own inalienable responsibility; therefore, the caregiver develops resources and strategies perfectly aligned and adapted to the needs of the dependent person.

***C5:*** “…*I saw it as an obligation [to take care of his wife], of course. No, no. It would have been selfish of me to take her to a care facility. This has happened… and I turn back on the whole matter… come on*!”

***C8:***“[…] *caring never ends. I could die first and this man would continue like this. And what a job I’d leave for my son… I do not want my son to carry this burden. I feel that I am indispensable for him. I barely think about him*”.

A great fear of not being able to offer this care in the future is detected in most of the participants´ discourse. Spouses are afraid of the stroke survivor being absolutely vulnerable if they are unable to offer their care.

***C1:*** “*If something were to happen to me, I would rest. I would have a load off my shoulders, but she would still be here, you see? And then… I would like my wife to die before me, even if a little earlier. To have one less problem, so that she would not have any problems. That is one of the greatest fears I have since this happened. It is better if she passes on first… more or less at the same time, so we do not have problems. I don’t want to leave her desolated here*”.

Second, a*ttachment and persistence in care* refers to a resistance to detachment from care provision. Interviewees take a clear stand against care facilities, preferring to take care of the person in a home environment. Despite home care is being understood as the logical, appropriate and correct option, caregivers recognise the need for partial support to act as pressure relief, so that they can contain and minimise their propensity to give up. This support would involve s*hort breaks as*: two or three hour breaks for leisure and recreation activities or to perform daily living activities away from home (change of location), backed by support from social networks, dependency aid and enhanced dependent autonomy.

***C1:*** “*I take care of her. The thing is, I’m looking for five days, so I can breathe a little and I know that of course the three of us will be together in Córdoba, if we finally go. Look, there’s a passage in Matthew that says ‘come sweet cross’ or something like that. And this is a cross to carry no matter how you take it, do you see? Whether you work it out or not, this is a burden. And it has turned my life upside down till that day*”.

The s*hort breaks* for breathing also refer to places of refuge where caregivers can recover their original/previous identity. These spaces are usually located at home. Caregivers designate a place, room or corner where they reconnect with themselves through short leisure activities or in the context of rest/sleep (the vast majority of couples no longer sleep in the same bed to make it easier for both to rest/sleep).

***C2:*** “*When everyone is already going to bed, I need to be on the couch in the lounge, spend a time just by myself… relaxed with my thoughts… and once I see that I am relaxed and feeling well, I go to bed*…”

### Breaking the couple’s life together

The second issue that aims to give some light on the experience of couples when one takes care of the other is the idea of *breaking the couple’s life together*.

Stroke is considered a relationship disruptor and it makes the caregiving spouse to see the dependent spouse as *someone else*; someone who is different to the man/women he/she was before the stroke (either due to physical, cognitive, emotional, behavioural or temperamental changes).

***C2:*** *He is there … but he is not the same. He has changed. We don’t talk like we used to… I don’t know if it’s because he’s not all there or because he’s not interested… he’s not himself*.”

***C15:***“ […] *and I said, these people have not seen my husband, what he was like before. There’s no point of comparison between what he is now and what he used to be. He’s a completely different person […] Like I say, I have a man at home but I don’t know where my husband is […] my whole lifetime with him, and the man who used to be my husband is gone, all gone. There’s nothing left*.”

The caregiving spouse also undergoes a major change, taking on the predominant role of caregiver and leaving other roles that were part of his/her identity before the stroke. They also stop being who they were and become *someone else* who will have to struggle to reconnect and build new attributes into his/her new identity.

The same thing happens with the couple (both spouses) that ceases to be what they were to *be something else* on which both must work. This links to the prior step of r*econnecting* with the other person in an exercise of interpersonal knowledge. In this process of reconstructing the couple’s life together, the quality of the relationship prior to stroke carries a special weight, either as a positive or as an adverse element. Therefore, a couple that worked things out and functioned previously well (in terms of listening skills, giving and receiving advice, offering guidance and help, trust, security, respect and contribution to the other’s growth) finds it easier to establish a healthy caregiver-care recipient relationship. They re-build their life as a couple, knowing each other deeper (what some participants mention as knowing *the essence of the other*) and establishing new types of communication, metaphorically expressed as “e*motional communication*” by participant coded with number 1 (C1). *Emotional communication* refers to non-verbal communication using facial and corporal expression, touch and the way of looking at each other. Furthermore, messages are focused on emotional feedback mainly empathy, compassion and trust.

***C1:*** “[…]*so that limits a lot. It is a very feeling-oriented, emotional type of communication. I touch her and ask her how she is doing or what is happening but without uttering a word, you see? I only write (because miraculously she can read) or we say it at an emotional level. With hugs, kisses and caresses. That method worked for us. I use written communication, and she communicates with caresses, questioning looks and smiles, we give each other a hug, a caress, and so for 24 hours a day. If there is something positive from this situation is that our union has deepened, we’ve grown closer, closer in our sadness, because it’s thanks to this dependence and affection that we’ve forged a more close-knit relationship*.”

In contrast, a neglected or dysfunctional couple before the stroke is at risk of developing exhaustion in the caregiver role and may even go through a breakup (with this new relationship defined by interviewees as a *sibling*, *flat mate* or *cousin* relationship).

***C2:*** “*My marriage… we are like brother and sister… there is no marriage as such. It as if we were siblings […] I chose to take care of him… what I never thought possible is that he would forget I was his wife*.”.

***C12:*** “…*He sees me as a sister or a mother—not as his partner—, and only occasionally he’ll tell me, ‘but, hey, you’re my wife*!’

### Going through the (non-) loss alone

The survival or *non-loss* of the person who suffered a stroke (understanding loss as death after stroke) and the beginning of care involve a number of *losses* for the caregiver (loss of independence, loss of future expectations, loss of motivation, etc.). However, it is, above all, the loss of the other in certain relational aspects, which gives the caregiver a great feeling of loneliness, even when living near the dependent.

***C2:*** “*The feeling is that I miss my husband. I need a shoulder to lean on, someone to talk to, and someone who comforts me. I just don’t have that… maybe these are moments of loneliness… Now, for example, when my mother died I even told myself, ‘now you’re on your own’… I don’t know, it’s a feeling that I miss my husband, I miss someone to lean on, someone to talk to, someone to comfort me and… and I just don’t have it… Yes, I have my daughters, but*…”

***C15:***“[…] *what I miss most is his protection, as it were, the feeling of being protected, sheltered, by your partner*.”

The spouses also feel an intense sense of loss sometimes expressed as t*he other acquired disability* (handicapped or limited life with lack of possibilities to enjoy and flourish). This feeling is privately managed without caregivers being able to share the burden with the dependent or other people in the environment. As it is interpreted by some of the caregivers, occasionally, the dependent considers him/herself the only one who is in need of care (exhibiting *selfishness* and being blind to the caregiver’s losses, needs and demands). What´s more, attending to the family-relatives and community support network, most of the participants have the sense that no-loss (the survival, not death of the person who has undergone stroke) is over the experience of daily-life losses and biographical disruption. As consequence, they live in an *infinite loneliness*, coated by a lack of understanding and avid emotional support.

***C14:*** “…*The loneliness is infinite. You feel so bad… I never wish for his death… but I do wish for mine… I’d like to disappear and let that be the end of it… we’ve made it thus far, and I can’t go on*…”

***C17:***…“*I say yes, this has happened to you, but don’t you realise, are you not aware that involuntarily this has affected me? No, he does not want to see it. Even when I have had a low moment when I have cried, he tells me ‘I don’t understand why you’re crying. Why would you cry?’ With what I’ve got going on, you’re going to cry on top of that?’ Do you understand? I can’t be neither fragile nor the martyr in all this* […].”

## Discussion

Comparing the findings of our study with those of other authors, we can say that spouses who care for a partner after a stroke are also intensely altered. This alteration results in a loss of earlier life—as reflected in the expressions of *losing the life that once was* [41] and *lives turned upside-down* [20]—and the adoption of a new vocation in the role of caregiver. As Young et al. mention in their study, the grief response can be strong so that a comprehensive caregiver assessment is necessary to discover the meaning of the experience as well as the adjustment phase of the caregiver [42].

The loss of independence, autonomy and ability to plan daily living, normal patterns and relationships as well as the loss of certainty about stroke event and its consequences in a medium-long term and the loss of self confidence in coping with this, result in spouses negative psychological experiences [43].

Regarding the latter, previous research indicates that approximately one year after the stroke, roughly half of the spouses experience high levels of caregiver role fatigue, with depressive symptoms and intense dissatisfaction with life [44]. Confirming our results, other authors refer to the lives of caregiver partners as restrictive and difficult [44], highly conditioned by apathy and with great resentment towards the required changes in their own lives because of the couple’s health problem. By contrast, the literature also refers to care-related positive experiences, which are eclipsed at times by the downsides but do exist as well. The identification and enhancement of these positive experiences improves caregiver’s life satisfaction and quality of life [45].

Roles’ changes are considered as a burden, emotionally, mentally and physically by spouses [46]. They are afraid of not being themselves anymore, and the fact of being in charge of all tasks alone intensely scares them. Constant availability and lack of knowledge are also contemplated as overwhelming. Attending to this last issue, many caregivers highlight the fact that no one has prepared them for caring neither for stablishing new ways of communication with the stroke survivor resulting in an enormous exhaustion [47]. Some consequences of this overwhelming experience are: lower vitality (fatigue and physical exhaustion), higher mental (anxiety and depression) and general health diseases incidence (sleep deprivation, back pain, headaches…), and social disfunctioning, which altogether result in a lower quality of life [9, 43].

In terms of couple biographical disruption, different authors, in the same line as our findings, express it as *being trapped in a drained marital relationship* [45] and a move away from the other person, considered *different and unrecognizable* [48]. Sometimes people express the feeling of living with another person, and feelings of alienation increase over time [45, 46] where a sense of loss in mutual understanding turns married life into a mere caregiving relationship [11].

Apart from the physical, functional, behavioural, role performance and psychological/emotional changes that could condition this non-recognition or rejection, McPherson et al. [49] describe the importance of striking a balance between giving and receiving; in other words, alignment between the dependent person’s and the caregiver’s concept of care is necessary for ensuring a healthy relationship. In addition, Bäckström et al. [50] added the need expressed by caregiver partners to receive love, empathy and emotional support from the dependent person as a necessary core element to sustain a marital relationship and a bidirectional, balanced care.

Kramer [51] connects the earlier marital relationship with the way a couple copes with the new caregiving relationship, identifying risk factors in advance. Thus, conflicting or damaged relationships place the caregiver in an especially vulnerable position where fatigue can arise in the context of a marital relationship that may have been non-existent prior to stroke. Solid interpersonal relationships (“closed-knit family”) are recognised as very powerful coping resources [52], although this does not mean that they are immune to the continuous risk of deterioration; being a constant concern among caregiver partners [53].

Last, regarding *Going through the (non) loss alone*, other authors analyze the great feeling of "loss" and the experience of loneliness. This loss is described in different dimensions that go from the purely functional aspects (loss of leisure time and freedom) to relational and emotional aspects (communication, support from the partner) [54].

### How should the findings be used to influence practice?

Taking together the present results, the contributions of other researchers, the methodological limits previously mentioned in the methodology section, and the characteristics of the field/sample of this study into account, the authors suggest the following recommendations to guide -caregiving spouses in role both transition and the biography restructuring process.

A family-centred approach to guide nursing care starting with a complete family assessment as it is proposed by Clarkin, Frances and Moodle in 1979 would be appropriate [55]. *Interrupted family process* may be considered the primary problem that influences others such as *situational low self-esteem*, *hopelessness*, *caregiver role strain* and *anxiety*.

Care planning for addressing these problems involves designing programmes combining both individual psychotherapy and couple therapy. As Luker et al. systematic review advices, caregivers demand counselling for creating reflection opportunities to develop coping strategies [47]. Using the spouses´ individual psychotherapy approach, suitable nursing interventions could include the following: anxiety reduction, relaxing therapy, problem solving training, cognitive restructuring, counselling and group support.

Couple (family) therapy would be tightly linked with assertiveness training, limits and common goals establishment, and role strengthening. Furthermore, family support is necessary in terms of increasing aid resources and problem solving.

These interventions should be applied as soon as possible once the stroke survivor is stable and the caring role is going to be transferred from nurses to spouses. Family assessment is the core element of the care plan and would be guiding nursing diagnoses and interventions as it improves.

Future research should focus on evaluating the discussed care interventions, so that clinical practice guidelines can be developed based on evidence and clear proposals to guide individualised care planning.

## Conclusions

After stroke, spouse caregivers have to cope with different losses, life changes and new challenges. The loss of previous roles and, as consequence, one’s identity is decisive in the transition to caregiving and dependence. Nurses should address the process through a comprehensive and integrated approach focused on transition care.

Care resources must be adapted to the interpretation that the spouses have of their caregiving role and their relationship with the different components of the caregiving process, taking in account that caregiving is an exhausted experience lived in loneliness.

The couple assessment is essential to identify vulnerable situations, and to guide the implementation of interventions promoting the couple´s biography (self-knowing for further knowing and accepting the other).

Making losses and their consequences, visible to health professionals, relatives, community and society may contribute to spouses´ experiences in being understood and consequently, to spouses’ developing appropriate roles as caregivers.
